# Rethinking crossover and recovery in eating disorders through a dynamic and value-sensitive framework

**DOI:** 10.1007/s11019-026-10328-4

**Published:** 2026-02-18

**Authors:** Davide Serpico, Valentina Petrolini, Silvia Camporesi

**Affiliations:** 1 Department of Philosophy, University of Milan, Via Festa del Perdono 7, Milan, 20122 Italy; 2 Department of Philosophy, University of Bologna, Via Zamboni 38, Bologna, 40126 Italy; 3 Centre for Biomedical Ethics and Law, Department of Public Health and Primary Care, KU Leuven, Kapucijnenvoer 7, Leuven, 3000 Belgium

**Keywords:** Eating disorders, Diagnostic crossover, Recovery, Identity, Values, Epigenetic landscape

## Abstract

Eating Disorders (EDs) raise significant challenges from a diagnostic and nosological perspective. Much of this is due to the extensive overlap among diagnostic criteria, with symptoms being shared by several conditions and subtypes. This nosological uncertainty is further exacerbated by two additional features of EDs, which will be the focus of this paper, namely diagnostic crossover and recovery. First, patients who acquire or lose one or more symptoms over time (symptom shifting) often transition to a new diagnostic category (crossover). Second, researchers working on EDs have recently underscored a problematic lack of inclusion of patients’ perspective on diagnostic and recovery processes, which results in an incomplete understanding of key aspects of EDs. Drawing on theoretical frameworks and concepts from Dynamical Systems Theory and epigenetics, we present a dynamic characterization of EDs that allows us to tackle the challenges of crossover and recovery. In our framework, different conditions represent robust endpoints in individuals’ developmental trajectories that are nonetheless flexible in certain circumstances. Thinking about EDs in diachronic terms also prompts us to significantly reframe our notion of recovery, not so much as the return to health but as the generation of future healthy trajectories, to be pursued in compliance with patients’ self-perception and aims. Indeed, the key role of patients’ values in determining their future trajectories testifies how psychiatric categories are not merely descriptive but constitutive, influencing both self-understanding and clinical practice.

## Introduction

Feeding and Eating Disorders comprise a heterogeneous class of diagnostic constructs, including Anorexia Nervosa (AN), Bulimia Nervosa (BN), and Binge-Eating Disorder (BED). Each of these categories also includes severity subtypes – such as “mild”, “moderate”, and “in partial remission” – that have been expanded and specified in the latest revisions to the *Diagnostic and Statistical Manual of Mental Disorders*, DSM-5-TR (APA 2022). Similarly to what happened with other DSM conditions, the transition to more recent editions of the diagnostic manual has brought about a progressive pulverization of categories (Stanghellini et al. 2021). This translates both in the addition of novel conditions (e.g., Binge-Eating Disorder) and in the relabeling of previous ones – e.g., Feeding Disturbance Disorder has become Avoidant/Restrictive Food Intake Disorder (ARFID).^1^

The structure and nosology of the DSM notoriously raise conceptual as well as practical issues that have been discussed by philosophers of psychiatry and clinicians alike (Stein et al. 2024; Kincaid and Sullivan 2014; Tabb 2019; Tsou 2016). As we show in this paper, many of these issues also affect the current classification and understanding of Feeding and Eating Disorders (EDs henceforth). Some of these problems are very general in nature and mostly concern the way in which the DSM is constructed, that is, as a *polythetic* and *descriptive* tool to support diagnosis and classification (Fellowes 2022).

First, the polythetic structure of the DSM implies that diagnostic criteria often allow individuals to cross the clinical threshold(s) in different ways. This in turn creates a situation where people can receive the same diagnosis without sharing any symptoms (Olbert et al. 2014), or – when disorders are not described polythetically – where different diagnostic categories significantly overlap in terms of individual symptom items. This casts doubt on whether we are identifying sufficiently homogeneous conditions that are useful for successful inferences and targeted therapies.

Second, the DSM descriptive approach prompts psychiatrists to tackle diagnosis ‘pragmatically,’ by relying on the symptoms that cause the greater amount of suffering or on those that better respond to treatment (Maj 2011; Fairburn and Cooper 2011). This has been effectively summarized by Kendell’s in his *dictum* on clinical utility: “All diagnostic concepts stand or fall by the strength of the prognostic and therapeutic implications they embody” (Kendell 1975: 40). Yet, such an emphasis on utility frequently conflicts with the scarcity of compelling etiological explanations, which renders the very distinction among psychiatric conditions subject to a certain degree of arbitrariness (Maj 2005). This is further complicated in cases of comorbidity, where different clinical entities within the same patient interact with one another in complex and unpredictable ways (Petrolini and Vicente 2022).

Some of these general problems with the DSM approach also affect our current understanding of EDs, especially at the level of identifying homogeneous clinical clusters. Indeed, several researchers working on EDs have recently called attention to the fact that some key features remain unresolved, with patients exhibiting very different symptoms over their lifespan (Stanghellini et al. 2021), diagnostic boundaries being highly fluid (Hay 2023; Milos et al. 2005), and recovery rates being extremely low (Breithaupt et al. 2022). Moreover, recent discussions have underscored a problematic lack of inclusion of patients’ perspective (Jaiprakash et al. 2024), which results in an incomplete and insufficient understanding of key aspects of EDs unrelated to eating behavior – e.g., desire for control, or pleasure derived by purging behavior (Ralph-Nearman et al. 2021; Christian 2024).^2^

In this paper, we consider these challenges in the context of EDs by showing how first-person perspectives on these conditions, together with considerations inspired by emerging dynamical models, can productively inform the diagnostic process as well as conceptions of recovery.

Before doing that, in the next section we delve deeper into some more specific features of EDs that make it challenging to undergo this much-needed rethinking within the DSM framework. Then, in An epigenetic framework for eating disorders, we present a dynamic characterization of EDs, where various disorders represent robust endpoints in individuals’ developmental trajectories that are nonetheless flexible in certain circumstances. Thinking about EDs in dynamic terms will allow us to address two key challenges emerging in the clinical literature, namely, diagnostic crossover and recovery. Additionally, our framework will show how patients’ experiences, as well as their values and self-identity perception, play a key role in determining their clinical path. This corroborates the idea that psychiatric categories are not merely *descriptive* but *constitutive*, influencing both individuals’ self-understanding and clinical practice.

In the conclusion, we consider implications for potential revisions of the DSM taxonomy and the role patients’ values may play in therapeutic settings. A diachronic and processual understanding of EDs prompts us to significantly reframe both diagnostic crossover and the notion of recovery in more dynamic terms. For crossover, we show how past trajectories constrain the future development of individuals (*canalization*), which potentially explains why some diagnostic transitions are more likely (or unlikely) to happen than others, depending on how early in life one has developed the relevant condition. For recovery, we propose a significant reconceptualization by moving away from the idea of ‘returning to health’ towards a notion of recovery as the *generation of future healthy trajectories*, to be pursued in compliance with patients’ self-perception and aims.

This general reframing also allows us to make some progress in the understanding of EDs nature and trajectory, especially in the relationship between identity and diagnosis. While we tend to think about EDs as somewhat episodic conditions, similar to mood disorders, their depth of canalization resembles more closely neurodevelopmental conditions (such as autism or ADHD), for which it makes little sense to talk about personal identity as separated from the diagnosis. Given their early age of onset – i.e., late childhood through adolescence – and subsequent chronicization (see The challenges of eating disorders taxonomy), the trajectory of EDs also shares important similarities with schizophrenia, which is hardly reversible and profoundly changes self-perception and self-understanding. Drawing on qualitative data and first-person accounts, combined with our dynamic and epigenetic framework, we show that EDs display an important identity component that has to be taken seriously in reflecting on processes such as diagnostic crossover and recovery.

## The challenges of eating disorders taxonomy

EDs raise significant challenges from a diagnostic and nosological perspective. As we explain above, many of these challenges are not specific to EDs, but rather connect to the general approach endorsed by the DSM, which privileges *symptom-based* descriptions focused on observable behaviors at the expense of etiological explanations. Yet, some of these challenges are connected to the specific way in which EDs are classified. One of these challenges arises from the fact that – unlike the majority of DSM conditions – EDs are described non-polythetically, i.e., all the criteria have to be met for the diagnosis to be assigned. As a consequence, in the DSM descriptions of EDs we witness an extensive overlap among diagnostic criteria, with symptoms such as “extreme weight control behavior” or “self-induced vomiting” being shared by several subtypes. While polythetic disorders tend to give rise to a heterogeneous distribution of features within the same condition, disorders that are described non-polythetically result in a similar distribution of features across different conditions. In the case of EDs, the fact that several criteria are shared by AN and BN results in the possibility of two individuals receiving one of these diagnoses while exhibiting a similar clinical profile. For instance, more than half of individuals with BN or with purging disorder also meet the criteria for atypical AN (Forney et al. 2024). As a result, it is often difficult for clinicians to understand whether these diagnoses reflect the presence of distinct clinical entities or rather refer to multiple manifestations of a single clinical entity (see Maj 2005). Another unfortunate consequence concerns *nosographic pulverization* (Aragona 2009; Stanghellini et al. 2021), which creates a plethora of sub-syndromes described with reference to the main syndrome minus one or two features: e.g., in Atypical AN, all the criteria for AN are met except that despite significant weight loss, the individual’s weight is within or above the normal range. It is therefore unsurprising that the demarcation between EDs has been recently identified as a research priority area (Becker et al. 2020; Hay 2023).

This diagnostic and nosological uncertainty is further exacerbated by two additional features of EDs, which will be the focus of this paper, namely, *diagnostic crossover* and *recovery*.

Starting with diagnostic crossover, as we mentioned above, several diagnostic criteria are shared by more than one ED. Yet, given the lack of comorbidity among EDs, patients who acquire or lose one or more symptoms over time (*symptom shifting*) often transition to a new diagnostic category (*crossover*). Crossover appears to be particularly common during the first 5 years following diagnosis, where crossover rates are around 34–36% from AN to BN and around 14–27% from BN to AN (Tozzi et al. 2005; Eddy et al. 2008; Schaumberg et al. 2019).^3^ Crossover between subtypes, especially among restrictive ones, is estimated to be even higher. For example, Breithaupt and colleagues (2022) found a high likelihood of transition from binge-eating/purging to restricting (72%), as opposed to the transition from restricting to binge-eating/purging, which was around 23%.

A large-cohort study by Schaumberg et al. (2019) evaluated the rates of diagnostic stability, crossover, and remission in a large sample of treatment-seeking individuals born in Sweden and compared rates of transition and stability across ED diagnoses at annual treatment visits. One of the interesting findings of this study was that transition from BN to AN and vice versa reduced the likelihood of remission for both groups (independently of the initial diagnosis). This pattern confirms previous literature (Eddy et al. 2008) indicating that diagnostic crossover is an indicator for poor prognosis.

Although the underlying causes of these phenomena are unknown, it is interesting to note that specific subgroups of patients appear to shift more frequently.^4^ In a retrospective longitudinal study involving data for 9,798 patients extracted from different databases, Garke et al. (2019) identify a sizable subgroup of 422 “shifters” (13% of the sample). While some of these individuals seem to fluctuate quantitatively between different aspects of the disorder – e.g., decrease in one symptom follows increase in another within the same category – for others the decrease of symptoms co-occurs with the increase of other problematic behaviors such as substance abuse. Breithaupt et al. (2022) also show that patients diagnosed with AN or Atypical AN tend to fluctuate or shift more across symptoms than patients diagnosed with ARFID, who tend to be more stable in their diagnosis.

One may wonder whether etiological data could facilitate drawing clearer boundaries between various conditions. Unfortunately, studies on risk factors suggest a high degree of overlap between ED subtypes at the etiological level, too. Candidate-gene and genome-wide association studies, for instance, provided limited evidence of robust associations between specific EDs and genetic variants (Bang et al. 2023; Mayhew et al. 2018; Zerwas and Bulik 2011). Moreover, many studies revealed genetic correlations between EDs and other conditions, indicating etiological overlap among fairly different psychiatric phenotypes like AN, BED, bipolar disorder, ADHD, obsessive-compulsive disorder, Schizophrenia, Bipolar Disorder, and Major Depression (Burstein et al. 2022; Bang et al. 2023; Hübel et al. 2021; Yilmaz 2020).^5^

The fluidity of diagnosis seems to be accompanied by equally relevant forms of *barriers* between diagnoses and, interestingly, different paths to recovery.

The literature on recovery dates back to the late 1990 s, where it emerged mostly in relation to the disability movement, the recovery movement (Harper & Speed 2014), and person-centered psychotherapy (Rogers 1967). Yet, a consensus on the definition of recovery in psychiatry has been difficult to achieve (Noordenbos and Seubring 2006; Noordenbos 2013; Stockford et al. 2019). One key issue concerns the fact that recovery in EDs can be conceptualized in very different ways, ranging from *narrow* views focused on weight restoration and the lack of relevant symptomatology to *broader* views emphasizing improvement in mental health outcomes and the possibility to live a meaningful life compatibly with one’s condition (Le Boutillier et al. 2011; on EDs specifically, see LaMarre et al. 2024; Khalsa et al. 2017). Stockford et al. (2019) stress the fact that narrow notions of recovery often endorsed in clinical studies are responsible for the current lack of a comprehensive and long-term approach on EDs. As a consequence, “it is not possible to reliably predict which patients will recover and which will develop chronic AN” (Stockford et al. 2019: 344).

Narrow definitions turn out to be problematic for at least two reasons. First, recovery studies rarely take into account diagnostic crossover (Khalsa et al. 2017; Mortimer 2015), making it likely for one patient to “recover” from one ED only to be diagnosed with another. This issue is particularly relevant given the high rates of crossover between EDs subtypes. Second, longitudinal studies, such as Breithaupt et al. (2022), show that rates of recovery tend to be dramatically low in the case of EDs (0.00% for AN-binge/purge subtype, 0.08% for AN-restricting subtype, and 0.36% for ARFID).^6^ In this respect, as we emphasize in the next section, recovery may be particularly difficult to define due to the nature of the disorder itself. Similarly, high rates of relapse may be a byproduct of our scarce understanding of EDs trajectory (Khalsa et al. 2017).

In recent years, an emerging body of qualitative literature has examined the ways in which patients diagnosed with EDs understand recovery. What is common to all these studies is that patients see recovery as a unique and subjective process, often non-linear, involving far more than weight restoration or behavioral change. Patients themselves often reject the narrow view in favor of a more holistic perspective where recovery goes far beyond the idea of not crossing the relevant DSM diagnostic thresholds. This research also highlights how patients’ values, self-perception, and life experiences can shape their clinical trajectories, preventing them from accepting – more or less explicitly – diagnostic transitions from one ED to another or even embracing treatment and recovery. As we shall discuss in An epigenetic framework for eating disorders, patients often adopt a hierarchical and value-laden language, with conditions like AN being described as more desirable than BN (Christian 2024; Eli 2018; Frey 2020; Mond and Arrighi 2012). For similar reasons, some patients show forms of resistance to recovery or reject diagnostic changes (Aviv 2022; Frank 2013; LaMarre et al. 2024; Jenkins and Ogden 2012; Mortimer 2015, 2019; O’Connell 2023; Tan et al. 2003).

Before considering these data, in the next section we introduce a theoretical framework that better captures the dynamic nature of EDs and thus better accounts for diagnostic crossover and a process-oriented view of recovery. Notably, such a framework also does justice to first-person accounts of people diagnosed with EDs and better fits the way in which they describe their experience.

## An epigenetic framework for eating disorders

Symptom shifting, diagnostic crossover, and recovery share something important from a conceptual viewpoint, that is, all of them can be adequately captured only by moving away from a *snapshot* view of EDs. As Fairburn & Cooper put it:

It is all too easy to get carried away with nosological niceties […]. Doing so can divert attention from […] the uncomfortable truth that eating disorders are not stable. […] Eating disorder diagnoses are *snapshots in the course of an eating disorder* (Fairburn and Cooper 2011: 9; our emphasis).

Notably, this static view seems to be championed by the very way in which the DSM is organized. Indeed, although some temporal markers are present in the description of EDs – e.g., “extreme weight/shape concerns over the past 4 weeks” – the bulk of the descriptions focuses on individual symptoms (e.g., binge-eating) and behavioral indicators (e.g., laxative misuse), more so than on the observation of how the disorder evolves over time.

By contrast, we propose adopting a *diachronic view* of EDs that turns more explicitly towards observing changes over longer timeframes. Drawing on theoretical frameworks and concepts from Dynamical Systems Theory and epigenetics, we make the case that various types of EDs are characterized by different developmental histories. This interpretation, as we will show, is particularly effective to characterize dynamic changes in health and pathology such as symptoms shifting, diagnostic crossover, and recovery.

The use of dynamic models is rapidly increasing in many medical areas. Models in oncology, for instance, describe cancer as the transition from one robust cell state to another (Huang and Kauffman 2013; Davila-Velderrain et al. 2015; Moris et al. 2016). Similar ideas are emerging in psychiatry and endocrinology (Carhart-Harris et al. 2023; Demori et al. 2024; Olthof et al. 2023; Serpico and Petrolini 2023; Trefois et al. 2015). The common intuition behind these models is that humans, as living beings, are complex systems characterized by a constant pursuit of stability through continuous adaptation to internal and external perturbations. This lifelong process was represented by Conrad H. Waddington (1957), the founder of epigenetics, as a landscape of alternative pathways determined by the interaction between an individual’s biology and environment.

**Fig. 1:**
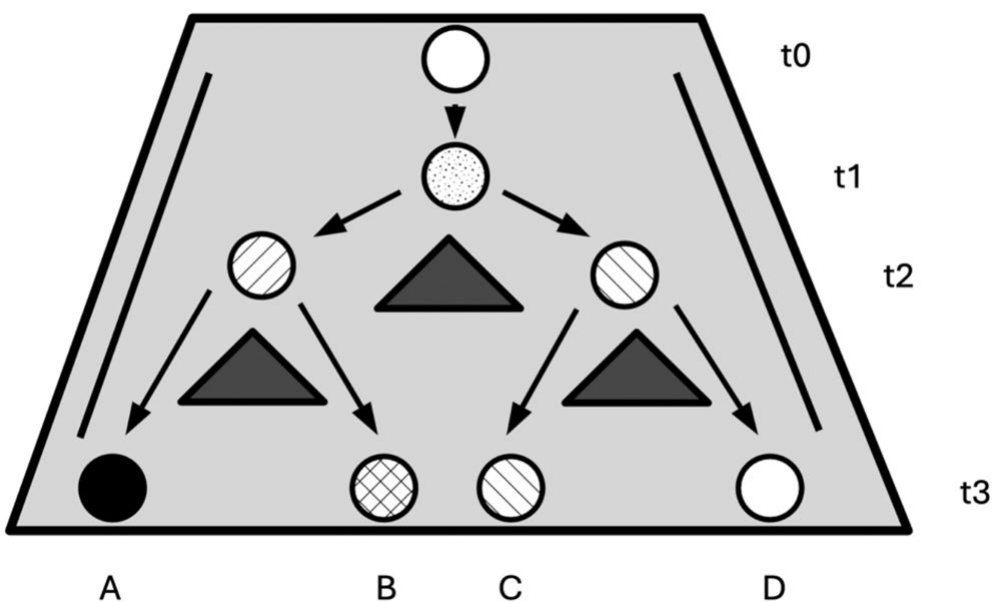
A representation of the epigenetic landscape. Each developmental pathway is associated to a robustendpoint (A, B, C, D) originating from bifurcation events at t1 and t2 (adapted from Waddington(1957). The strategy of the genes. London: Volume George Allen and Unwin)

In this popular representation (Fig. 1), each pathway originates from bifurcations in development (at t1 and t2) and is associated with distinct and robust endpoints (A, B, C, D). Such robust states, called *attractors* in the vocabulary of Dynamical Systems Theory, tend to exercise a stronger force that pulls other states towards them. Mental conditions, in our view, are therefore *discrete*, qualitative states that may be represented as such endpoints within this model.^7^

At the same time, development is a flexible process, which means that individual trajectories can change over time if subject to sufficiently strong perturbations, leading to dramatic physiological and psychological changes. This is particularly likely to happen at *developmental switch-points*: some periods in life are bound to make individuals more vulnerable to perturbations. This in turn can open the door for biological and environmental perturbations to generate *bifurcations* in development that affect the entire developmental trajectory – not just a single aspect of health – thereby causing cascades of events that canalize individuals into qualitatively different trajectories.^8^

A key aspect of bifurcations is that, in many cases, they make some trajectories *unavailable* or *inaccessible* until a new bifurcation originates. In this sense, developmental potential is constrained by previous paths, and at the same time, it narrows down over time, being channeled or canalized into robust pathways. This path dependency, as we shall see, is especially relevant for conditions like EDs because of their early onset and constraining force.

### Applications: diagnostic crossover

In this section, we analyze data from various sources – observational, molecular, and qualitative studies – that shed new light on the bio-psychological processes underlying diagnostic crossover in EDs. As we shall see, first-person perspectives are particularly interesting to get a glimpse of the powerful role played by patients’ values in shaping clinical and institutional trajectories. In a sense, EDs represent a clear case of *reflexivity*, highlighting how the very act of classifying people (e.g., psychiatric diagnoses) influences them, inducing “changes in self-conception and in behavior of the people classified” (Hacking 1995: 370).

As we mentioned in The challenges of eating disorders taxonomy, observational studies highlight that EDs follow specific temporal dynamics in terms of progression, particularly when we look at the transition from one ED to another. A potential explanation involves the sequence of events connecting various EDs, leading to symptom shifting and, subsequently, to diagnostic crossover. According to cognitive-behavioral theories, for instance, two psychological mechanisms underlie restriction: a need to feel in control, pursued through eating, and over-evaluation of body shape and weight (Fairburn and Harrison 2004). The consequent restriction is *highly reinforcing*, resulting in a perpetuation of the *vicious cycle* and facilitating the onset of other maintenance factors such as social withdrawal, higher binge-eating frequency (promoted by restraint), and negative effects of the loss of control (i.e., secondary negative self-evaluation and need for an even stricter self-control).

In light of similar data, Eli and co-authors (2015, 2022) argue that existing clinical models of EDs can be strengthened by including the experience of binge-eating as a potential disorder-maintaining factor. Cognitive-behavioral therapy is only “moderately successful,” and the authors suggest that this might be the case because “while this model accounts for the mood regulation effects of binge eating, it does not address patients’ valuation of binge-eating experiences” (Eli 2015: 5). We will return to this point below when we take a closer look at how patients’ values can affect the trajectory of EDs experiences.

At the molecular level, there is also evidence that environmental factors, such as nutrition and stress, can determine epigenetic changes that alter genetic expression affecting the long-term risk of developing different EDs (Campbell et al. 2011). For instance, Frieling et al. (2010) found that both patients with AN and BN show elevated expression of the dopamine transporter gene (DAT) and downregulation of the dopamine receptor D2 gene (DRD2). However, significant hypermethylation of the DRD2 promoter was only present in the AN group. Similarly, Hübel et al. (2019) highlight inconsistencies in epigenetic effects between AN and BN – DNA hypomethylation and hypermethylation, for instance, is present in AN cases with no significant difference in BN cases.

Both observational and molecular data therefore suggest the need for a time-sensitive perspective on EDs, one that pays closer attention to the lived experiences and values of diagnosed patients. What types of trajectories characterize different EDs, and why are some directions of crossover (or transitions) more frequent than others?

Thinking about EDs in dynamic terms allows us to see that some temporal dynamics are *more constraining*, which may make it harder to cross some borders in developmental trajectories. Patients’ history, in our hypothesis, determines the form the condition will take: in the epigenetic landscape metaphor, this translates into the valleys and the potential switch-points available to the individual at a given point in time. Assuming each trajectory in the landscape is a type of ED, the topology of the landscape illustrates that some valleys are *closer to each other*. The closer two trajectories, the more similar their symptomatic manifestations and historical development, and – as a consequence – the more likely they will be categorized as belonging to the same category or the easier it will be to transition from one to the other. A potential explanation of crossover, then, is that clusters of symptoms associated with different EDs reflect distinct trajectories, but these trajectories are sufficiently ‘close’ to each other (e.g., similar in terms of etiology, developmental dynamics, and symptomatic manifestations) to allow individuals to ‘cross the borders’, generating paths connecting two valleys in the epigenetic landscape.

Another interesting factor, revealed by qualitative data and first-person accounts, is that some *diagnostic barriers* could be also determined by patients’ perspectives on crossover as well as how they *value* potential diagnostic transitions. Previous studies show that perception of diagnostic crossover is deeply entangled with social contexts. Mortimer’s (2015, 2019) study, for example, suggests that patients with EDs often feel part of a community of like-minded people with whom they deeply identify. Loss of community membership through diagnostic crossover was thus often experienced by participants as *undesirable*. Hollie (pseudonym), one of the participants, uses the expression of “the rug pulled out from under you” to express how, for her, transition from AN to BN felt as “though the comfort, security and predictability of AN had suddenly and inexplicably been taken away” (Mortimer 2015: 50). She experienced this as “disorientating” and was left with intense feelings of “shame, guilt and failure” (*ibidem*). Another participant, Marianne (pseudonym), puts it in even stronger terms:

It’s weird to imagine your 12 or 13 year old self allowing that to happen. She’d be horrified. Absolutely. […] oh god this sounds so trite doesn’t it, but who I was back then, would, would be horrified, and there was so much pride attached to the strength of that.

Similarly, EDs are often discussed by patients through *hierarchical* language, with AN typically being described as more desirable and acceptable than BN (Mond and Arrighi 2012) – e.g., “a bulimic is an anorexic who failed, and I had no intention of failing” (Eli 2018: 164); “with eating disorders everybody kind of wants to be anorexic” (Frey 2020: 143). A recent study (Christian 2024) investigates the perspective of women who had experienced crossover from ANBP to BN and/or from BN to ANBP, confirming the previous study by Mortimer (2015, 2019). Participants experienced “diagnostic crossover as impactful, stigmatizing, and ultimately unhelpful to their treatment and recovery” (Christian 2024: 61). For some participants, the new diagnosis became a point of contention over what symptoms (e.g., BMI or disordered eating behaviors) are the most clinically significant (Christian 2024: 62). For others, the experience of crossover itself led to an intentional intensification of restrictive eating behaviors in order to regain the diagnosis of AN which was lost with increased BMI. De Moor and co-authors (2024: 349) have also pointed out how perceived stigma “from close others, society as a whole, or media may contribute to adolescents’ internalization of prejudice and discrimination regarding their diagnostic label.”

This is related to the experience of a diagnostic hierarchy where certain EDs – i.e., restrictive types – are perceived by patients as “better” or “more prestigious” than others. O’Connell (2023) further explores this idea through an autoethnography of her own AN diagnosis. Building on Brinkmann’s (2016) distinction between “being” and “doing”, O’Connell discusses the performative aspects of EDs diagnoses, whereby patients learn to “outwardly display their symptoms in recognizable ways in order to legitimize their need for care” (O’Connell 2023: 264). In other words, through the interaction with clinicians and other patients, many people diagnosed with AN learn new ways to “be” anorexic and to “do” anorexia within a clinical system. Hospitalized patients, for instance, often compete with other patients towards being the ‘best anorexic ever,’ and come to identify strongly with their AN diagnosis. These phenomena testify the symbolic power that psychiatric diagnoses exercise, in this case through the patient’s perception of belonging to an elite and exclusive group (see also Warin 2010).

As we mentioned in The challenges of eating disorders taxonomy, epidemiological data suggest that some directions of crossover (e.g., from AN to BN) are more frequent than others (Tozzi et al. 2005; Eddy et al. 2008; Schaumberg et al. 2019). If diagnostic trajectories were primarily or even exclusively guided by values, one may expect BN-AN transitions to dominate over AN-BN ones. Our characterization of EDs, however, involves the *interplay* between canalization effects and patients’ values, and thus may shed light on potential reasons for these findings, ones that suggest a discrepancy between self-narratives and biopsychological feasibility.

One of such discrepancies concerns the fact that – despite their heightened desirability – restrictive diagnoses seem to be more easily lost to crossover with respect to bulimic ones. In these cases, according to our hypothesis, crossover directions may be shaped by an ‘asymmetric attractor landscape,’ in which AN represents a high-control and harder-to-maintain state compared to BN. Relaxation of control – and thus AN to BN transition – may therefore be more frequent than its reinstatement because of behavioral and physiological canalization effects. Within this picture, for many patients, values shape self-understanding and symptom expression, acting as directional forces on canalized dynamics, but the direction of transition is also constrained by behavioral and physiological factors that may be partially outside of their control.

At the behavioral level, binge-purge routines form self-reinforcing loops. Breaking this loop to achieve stable restriction demands a radical behavioral inversion: transitions from BN to AN thus require sustained inhibitory control and tolerance of starvation. Given that both of them are physiologically and psychologically costly, BN to AN transitions might be harder to achieve, whereas relaxing restriction (from AN to BN) represents a smaller perturbation of the control system.

At the physiological level, sustained restriction requires a level of metabolic and hormonal adaptation that is physiologically hard to re-establish after recurrent binge-purge cycles. Starvation induces long-lasting endocrine and metabolic adaptations (hypoleptinemia, altered HPA-axis, dopamine and serotonin dysregulation) that, in many cases, may make AN robust and difficult to reverse (Hebebrand et al. 2022, 2024; Kosmiski et al. 2013; Sidiropoulos 2007). Yet, these adaptations may predispose to binge-purge episodes once restriction weakens. Conversely, BN’s metabolic and reward-system dysregulation may increase drive for consumption and reduce tolerance of hunger. Thus, even if AN is valued, the organism may resist the shift.^9^

In terms of patients’ values, individuals with AN who experience loss of control may reinterpret it as personal failure and engage in purging to compensate, facilitating a transition towards BN. Instead, patients with BN rarely sustain restriction long enough to develop full AN symptomatology. Social and clinical feedback (e.g., weight restoration goals, therapeutic framing) may also make transition to restriction less socially and medically viable.

In the following subsection, we take a similar approach towards discussing the notion of *recovery*, by drawing both on clinical studies and first-person accounts of ED patients.

### Applications: recovery

Although narratives surrounding recovery are ubiquitous in the medical sciences, a robust conceptualization of the notion of recovery in psychiatry has proved challenging to achieve (Harper & Speed 2014; Davidson et al. 2005). The idea that it should be possible to recover from a condition, at least in principle, obviously grounds a great bulk of medical research and treatment. For many conditions, both somatic and psychiatric, recovery is associated with the transition from a pathological state to a healthy one. Yet, our epigenetic framework shows that things are much more complex. In our view, individuals can achieve a healthy state according to standardized parameters and thereby cease to meet diagnostic criteria – e.g., by restoring their previous BMI in the case of EDs – but such a state should not be equated to a healthy state of someone who has never developed the relevant condition in the first place. The epigenetic component of the framework thus prompts us to significantly reframe our notion of recovery, by regarding it as the *generation of future healthy trajectories* as opposed to a *return to health*.

In this subsection we motivate our resistance to the – otherwise intuitive – idea that recovery consists in restoring a previous condition of health. First, we show that the notion of recovery may be difficult to pin down because we may still not know enough about EDs trajectories and their degree of canalization to establish what counts as being recovered. As we explain below, developmental switch-points lead to long-term, partly irreversible changes in epigenetic trajectories. Second, we explore how people’s values about their journey may feature into our conception of recovery itself.

As we mentioned in The challenges of eating disorders taxonomy, an emerging body of literature has investigated the lived experiences of ED patients. We draw on this material to garner deeper understanding on patient-centered notions of recovery. We also aim to contrast recovery narratives that promote individual approaches focused on symptom reduction only, while disregarding social and identity aspects that often connect diagnostic labels to the need for collective recognition. Similarly to the cases of crossover examined above, the interplay between experienced symptoms and perspectives or values endorsed by patients significantly affects conceptions of what counts as recovery and whether standardized pathways of recovery are valuable. We tackle these two aspects in turn, by first delving deeper into the implications of our framework and then moving to the analysis of first-person reports surrounding diagnosis and identity.

First, our framework can convincingly explain why the generation of future healthy trajectories in the context of EDs proves particularly difficult. A key point in this respect concerns the *early onset* of EDs. In our framework, canalization is a matter of degree: some phenotypic traits can be very plastic throughout life, while others gradually become so robustly entrenched that it is nearly impossible to change them deliberately. For example, traits like cognitive abilities and metabolism are quite plastic, as individuals can effectively change their future trajectories through behavior and lifestyle (Sauce and Matzel 2018; Serpico and Borghini 2021). Others, like neurodevelopmental conditions, are less flexible (Serpico and Petrolini 2023). Due to their multifactorial origins (Knopik et al. 2017), many mental disorders typically fall somewhere in between: they are less canalized than neurodevelopmental conditions, but can nonetheless be subject to canalization processes due to the interplay between biological constraints and environmental factors.

Importantly, whether and how much a condition is canalized depends on the genetic and biological mechanisms involved, but more crucially, on temporal factors including *when* the condition developed and *for how long* it has affected the individual. In some critical periods, certain events or experiences can trigger developmental switch-points, and the earlier this happens – and the more impactful such triggers – the harder it is to escape a pathological attractor.^10^ Data on the onset of EDs speak in favor of early and robust canalization. As Verschueren and colleagues (2020: 9) note, “As the age of onset indicates, adolescence and emerging adulthood represent crucial life periods in which individuals may become vulnerable to display eating symptoms or even an ED.”

For instance, in AN, the highest incidence rates are among individuals aged 14–19 years, with a peak at 14–16 years old (Javaras et al. 2015) and some patients – including Aviv (2022) – being diagnosed as children. Incidence rates for BN are slightly higher, with a larger group of patients (about 75%) with a mean age of onset of 16 years and a smaller group with an onset at 23–25 years (Stice et al. 2013; Volpe et al. 2016).^11^

To clarify further, let us consider some examples from a wider range of medical conditions. Although it is typically possible to recover from bone fracture, a bone that has been broken at some point in life looks different – e.g., on an X-ray rendition – from the same bone that never underwent the same trauma. Severe obesity is a metabolic condition that is even harder to reverse, especially if developed during childhood or if extending for long periods. Indeed, developing obesity involves profound changes in one’s metabolism, psychology, and behavior: chronic inflammation and calories imbalance have long-lasting, systemic effects, such as reducing one’s ability to use the stored fat mass for energy purposes and to metabolize sugar (thereby leading to insulin resistance and potentially diabetes; Rohm et al. 2022). All of this intertwines with psychological factors promoting a sedentary lifestyle and consumption of calorie-dense foods. Crucially, when individuals clinically recover from obesity – that is, when they manage to lower their BMI and cease to meet the diagnostic criteria for obesity – their metabolism will not be the same as that of individuals who were never obese. For instance, their metabolism will react differently to changes in diet and physical activity (Fothergill et al. 2016; Greenway 2015; Martin et al. 2022; Tareen et al. 2020).

Due to the canalization processes described above, along with their typically early onset, it is often difficult for an individual diagnosed with AN starting in adolescence to go above certain BMI values. In other words, their highly canalized metabolic process becomes incompatible with recovery narrowly defined.^12^ Our framework promotes a view that emphasizes the overall constellation of psychological and biological characteristics, more so than individual symptoms or indicators, thereby allowing us to meaningfully talk about recovery in cases where the individual’s behavioral and psychological functioning can be considered ‘healthy’ even in the absence of specific weight or BMI values. In the case of EDs, this implies moving beyond the notion of “weight restoration” or of being “weight recovered” (Hay 2023).

We now move onto exploring how a diachronic notion of recovery for EDs also satisfactorily accounts for the value and identity components of these conditions. As it is often the case with psychiatric disorders, symptoms and related experiences often become part of one’s personal identity, especially in conditions developed in childhood or early adolescence (Aviv 2022; de Moor et al. 2024; O’Connell 2023).

Qualitative studies consistently show that recovery in EDs is often understood by patients as a “process and as an identity journey” (LaMarre et al. 2024: 12), both in the context of relationships with others and within the social context more broadly. Aviv’s experience (2022), for example, sheds light on the process of recovery as the creation of a new trajectory, one where the person succeeds in re-orienting their life around a new identity, goal, or narrative. This process often includes the construction of new meaning and purpose, “a deeply personal, unique process of changing one’s attitudes, values, feelings, goals, skills and/or roles” (Anthony 1993: 527). Verschueren and co-authors (2024: 49) confirm these earlier qualitative findings “in which the recovery from an ED is described as a complex and challenging expedition, which involves letting go of the ED-identity and discovering a non-ED-centric identity.”

The early onset of EDs, combined with their relation to identity, makes it unsurprising that patients would find it effortful to build a “non-anorexic” or a “non-bulimic” sense of self. In other words, the ‘restitution narrative’ of recovery does not hold true for many of these patients because there is no prior identity to go back to (Frank 2013). This additionally suggests that a notion of recovery based solely on BMI values is bound to be insufficiently fine-grained to account for these cases. Moreover, our diachronic and epigenetic framework is well-equipped to describe cases of heightened vulnerability and higher risk of relapse following recovery, because perturbations within the epigenetic landscape (Fig. 1) may easily lead to the generation of new pathological trajectories. In other words, the creation of healthy pathways following deep canalization from an early age proves particularly difficult to achieve: the new pathways need to be built effortfully and tend to remain fragile over time.

First-person accounts are particularly effective in describing these challenges related to the recovery process. For instance, Mortimer (2015) talks about her nonlinear journey towards recovery being closely connected with a process of self-discovery which enables the “old ED-self to fade into irrelevance.” At the same time, she acknowledges the depth of canalization of previous life experiences, as the new self is shaped by past illness. As she puts it:

While full recovery often involves a radical dissociation from one’s ED-self, the new identity does not form in a vacuum but instead grows out of the wreckage left over from years of suffering from an ED (Mortimer 2015: 65).

Other qualitative data highlight ambivalent feelings towards the recovery process. For instance, some of the participants in the phenomenological study by Jenkins and Ogden discuss how “the AN voice and thoughts” caused them to question whether they could fully recover (Jenkins and Ogden 2012: 28–29). The complex interplay between different parts of identity features prominently in these reports, with some people describing recovery as a progressive distancing and rejection of the AN component, and others wishing to retain AN as part of their identity (Jenkins and Ogden 2012; Tan et al. 2003).

In this sense, recovery can be best conceptualized as a diachronic process where new paths are forged out of compromise and re-negotiation of different aspects of the self. The experience described as self-illness ambiguity (Dings and Glas 2020; Drożdżowicz 2023) also seems particularly relevant in the case of EDs, as many patients are concerned about the extent to which their personality traits are authentic or rather a symptom of their disorder (Tan et al. 2003; Hope et al. 2011). This is further complicated by the fact that some AN patients experience some of their symptoms as ego-syntonic and the very diagnosis of AN can be regarded as “a badge of honour” (Mortimer 2019: 370). Moreover, studies on binge-eating practices have uncovered the value that these experiences have for many patients, which are often regarded as ways to “fill up an existential emptiness”, as responses to deep emotional pain, or as a liberation or release following extended periods of self-starvation (Eli 2015; Eli and Lavis 2022).

The complexity of these experiences allows us to reframe the notion of recovery in connection with identity. For some patients, the disorder itself clearly constitutes a part of their identity – i.e., “an entity inside of themselves, inhabiting a part of their minds or bodies” (Tan et al. 2003: 540). Although some participants in the same study report perceiving this entity as being distinct and alien, others clearly struggle with the idea of being “recovered” and losing a large part of their personal identity (e.g., “‘I wouldn’t know who I was’ without the diagnosis”, Tan et al. 2003: 542). As we explain above, this is bound to be particularly difficult for those who developed AN in early adolescence and lack a clear sense of self to return to what could be perceived as an alternative to anorexic identity (see also Aviv 2022). As suggested by Mortimer (2019), the “anorexic personality” may emerge in the young patient precisely because it serves a *purpose*, and consequently the patient may worry that full recovery entails the loss of this personality, thereby requiring the formation of a new identity which may not be as functional to that purpose.

## Conclusions and implications

Scientific taxonomies, like psychiatry categorizations, are expected to support inferential and clinical reasoning as well as intervention planning – in our case, to inform therapeutic strategies. While our framework is primarily concerned with how to best conceptualize the nature and development of EDs, it has potential implications for two main areas of clinical research: first, what nosological structure would better fit the dynamic and processual features of EDs and, second, whether a dynamic understanding of EDs, complemented with patients’ first-person experiences, can inform value-sensitive approaches to therapy. While we do not aim to draw final conclusions on these central questions, this section offers some preliminary reflections on both points.

Starting with implications for nosology, our account of EDs suggests a potential rethinking of current taxonomic practices. In particular, it invites reflection on whether the rigid diagnostic boundaries employed by the DSM can adequately accommodate the temporal complexity of these conditions and heterogeneity within each category.

Two alternative taxonomic proposals, recently discussed in the clinical literature (Hay 2023), provide a useful starting point. The first, *less radical* approach involves reconceptualizing EDs along key trait dimensions, such as impulsivity-compulsivity and reward sensitivity (more adequate for patients with predominantly loss of control over eating and binge-eating behaviors) *versus* sensory hypersensitivity and perfectionistic control (for patients with predominantly restrictive eating behaviors). This would allow diagnostic categories to be restructured around traits aiming to capture patients’ profiles at the psychological and emotional levels, rather than behavioral symptoms. One additional advantage of this approach would be to include profiles that are currently not formally recognized as EDs (e.g., food addiction might be included with other disorders characterized by recurrent binge-eating, while orthorexia might be included with other restrictive subtypes).

The second, *more radical* proposal is to unify all EDs under a single diagnosis, with behavioral and cognitive specifiers capturing the heterogeneity of manifestations. This approach would be welcome for patients who crossover and “find it strange as to why their diagnosis is at one point bulimia nervosa but as little as half a year late may be binge-eating disorder, or who are told their weight is not low enough for anorexia nervosa” (Hay 2023: 843).

From our perspective, the less radical approach is broadly compatible with a developmental understanding of EDs: it acknowledges the limitations of categorical distinctions, based on the fluidity of EDs, while maintaining a degree of conceptual specificity that aligns with patients’ lived experiences and clinical needs, it is compatible with the view that different EDs are somewhat robust and qualitatively different, while at the same time explaining patterns of diagnostic crossover and symptom shifting. However, it may still fall short of accounting for cases where individuals transition between dimensions or occupy ambiguous positions – thereby highlighting a residual tension between dimensional and categorical aspects of psychiatry classification (see Banicki 2020; Haslam 2014; Keil et al. 2017; Phillips 2020; Serpico and Petrolini 2023). For example, patients with BN who experience high levels of dietary restrictions as well as loss of control over eating would not fit neatly into one or the other dimension.

The more radical proposal, while appealing in its acknowledgment of longitudinal instability and shared risk factors, faces the challenge of excessive heterogeneity: given the outstanding variability between EDs, individuals under the new ‘umbrella diagnosis’ might end up having very little in common, which is a contentious aspect of the DSM-5 dimensional description of autism, for instance (Petrolini and Vicente 2022). Without a principled account of what unifies all EDs under a single construct, such a revision of the EDs taxonomy would risk overlooking clinically meaningful distinctions – e.g., between restricting and binge-purging profiles – which may involve fundamentally different motivational dynamics, self-construal, and patterns of emotional regulation. This concern is echoed in first-person narratives discussed in An epigenetic framework for eating disorders, which often resist assimilating different ED subtypes into a common identity or trajectory.

Still, a modified version of this more radical proposal could find support in our framework, if reconceptualized not as a move toward diagnostic unification, but as a way of capturing the *emergent* character of EDs across different levels of explanation. On this view, the apparent diversity of ED behaviors – whether restrictive, compulsive, or binge-related – can be understood as different responses to similar underlying psychological, affective, or social disruptions. In other words, different behavioral responses could serve similar functions, in the context of an individual’s lifelong adaptation, or have similar origins in terms of patients’ history. Along these lines, Breithaupt and colleagues suggested that different ED manifestations can be considered “a single disorder with a course that can include longitudinal symptom variation, rather than separate illnesses” (Breithaupt et al. 2022: 721). This would be similar to what described in Petrolini and Vicente (2022) with respect to comorbidity in autism, where the resulting picture is not additive but emergent, reflecting complex interactions among genetic dispositions, developmental trajectories, socio-cultural environments, and self-related values.

While not offering a blueprint for immediate nosological revision, our framework recommends a hybrid approach that avoids the rigidity of current DSM categories, resists overgeneralization, and instead captures fluid yet distinct developmental trajectories of EDs. This perspective supports taxonomies that are flexible, temporally sensitive, and responsive to first-person meaning-making – a shift that is increasingly urgent in light of both empirical evidence and patients’ reported experiences.

Turning to clinical implications, as we explained in An epigenetic framework for eating disorders, patients’ values can significantly shape and constrain their experiences and course of illness. Specifically, the patient’s relationship to their ED – experienced as an identity, a set of symptoms to be eradicated, or something in between – may affect engagement with diagnostic labels, therapeutic interventions, and potential recovery paths. It seems to us that clinical practice must be attuned to these values to effectively navigate the often-conflicting roles that identity and diagnosis play in treatment.

For many patients, EDs are not merely pathological conditions but have become integral to their conception of the self. This identity is particularly pronounced in disorders such as AN, where restrictive behaviors are perceived as morally or existentially meaningful. However, when patients align with their ED as an identity, recovery becomes more difficult because the behaviors associated with the condition often represent ego-syntonic *ways of being* – they feel in harmony with the individual’s self-conception rather than at odds with it. For instance, patients may resist a diagnosis they perceive as ‘inferior’ or seek to remain within a ‘preferred’ diagnostic category, even as their symptoms shift. This resistance challenges clinicians to rethink how they approach treatment goals, recovery, and the therapeutic relationship.

In light of these complexities, we see two potential directions for clinical intervention, both of which are shaped by different interpretations of how an ethical relationship between clinicians and patients should be constructed in order to accommodate patients’ values and experiences.

The first is a somewhat *paternalistic* approach, which prioritizes the patient’s immediate safety, especially when EDs are life-threatening. In this approach, clinicians may take the position that, regardless of the patient’s self-conception or identity ‘attachment’ to the disorder, their survival is paramount. This approach would require treatment to shift the patient’s self-perception, even if this requires dismantling the deep sense of control and purpose that may be bound up with the disorder. While this approach has clear advantages in cases of imminent danger, it can be difficult for patients who consider it a part of their ‘authentic’ self: identity dissonance may emerge, making recovery emotionally painful and fostering resistance to treatment.

The second approach, which we may call *deontological*, takes even more seriously the patient’s autonomy and values. Here, treatment focuses on managing the symptoms rather than attempting to force a radical transformation. This approach is grounded in the understanding that EDs, being highly canalized, are bound to constrain development in a way that is similar to neurodevelopmental conditions or personality disorders. In all these cases, the condition can become deeply entrenched in an individual’s sense of self, especially when it emerges during formative periods of life such as late childhood or adolescence.

As a consequence, recovery from the disorder – understood as the complete eradication of the condition – may be unrealistic for many people. Instead, symptom management and harm reduction take priority, allowing patients to navigate their condition in ways that minimize physical damage, without necessarily requiring a full rejection of the identity associated with the ED. This approach is supported, for example, by Christian: “Person-centered recovery frameworks, where individuals are able to determine what outcomes are important in their own recovery journey, allow clinicians and individuals with lived experience to acknowledge the complexity of recovery and creates space for individuals’ autonomy” (2024: 65).

These two approaches are not mutually exclusive, and in practice, therapeutic flexibility is crucial. The dynamic model of EDs that we propose highlights the need for context-sensitive interventions that take into account one’s developmental history, their attachment to the disorder, and the degree of flexibility and canalization of the condition. For individuals who have experienced EDs for a prolonged time period, particularly those whose onset occurred during early adolescence or critical periods of identity formation, canalization effects may be so strong that shifting trajectories entirely may be neither feasible nor desirable.

Recovery, in these cases as in other chronic conditions, shall be understood as an ongoing, possibly life-long process of adaptation, without necessarily attempting to *revert* to an earlier, ‘healthier’ self. This requires a shift in focus from standard to person-centered recovery framework. For example, instead of joining the call for efforts to standardize definitions of recovery, LaMarre and colleagues suggest that recovery might mean “finding new ways of engaging with or interpreting this struggle [of being in the world with an illness]” (LaMarre et al. 2024: 11). They also strongly reinforce the message, put forward by others, that weight restoration is “not an adequate signifier of recovery”, and “invite further investigation into how recovery definitions themselves may be constructed in ways that reinforce particular ideas about whose bodies are deemed recovered or “healthy.” (*ibidem*).

To conclude, our model of EDs concurs with the view that recovery may involve not the eradication of the disorder, but the construction of a new relationship to it, one that takes into account the patient’s own values and the broader social and cultural factors that may shape their perceptions of health and disease. Notably, this process should be co-constructed by patients and clinicians, to avoid both paternalistic attitudes and self-managed approaches to therapy (see Chapman 2023). Recovery frameworks based on these insights have the potential to empower patients to define what “recovery” looks like for them, while also acknowledging symptom fluctuation and diagnostic crossover as part of an ongoing process.

## Footnotes

1 See Attia et al. (2013) for a history of the changes between DSM-IV and DSM-5. For more recent discussions that also take into account the most recent DSM revisions, see Walsh et al. (2023); Hay (2023).

2 The debate on the inclusion of patients’ perspective in nosological revision has been ignited by the neurodiversity movement with very interesting and impactful results in the last decade, such as the transition from a categorical to a spectrum view of autism (Amoretti et al. 2021; Fellowes 2023; Kapp and Ne’eman 2020).

3 A recent systematic review (Serra et al. 2022) estimates that about 41% of patients with restrictive-type AN transition to disorders involving binging and purging behaviors, often with a worsening of the clinical picture and long-term outcomes.

4 We propose potential explanations for the differential rates of crossover between EDs in An epigenetic framework for eating disorders.

5 Data on prenatal and childhood experience also show overlap in non-genetic risk factors (Daniels et al. 2023; Ozsvar et al. 2023).

6 For further studies on the chronicization of EDs, see Eddy et al. (2017) and Speciani et al. (2021).

7 The fact that mental disorders are discrete and qualitative does not imply that they are always stable. In our view, some mental conditions may be robust over long timeframes but still be particularly susceptible to being attracted by nearby states – be they healthy or pathological. When in the grip of such *brink states* (Petrolini 2021), individuals tend towards attractors in their vicinity, with risk factors facilitating the transition towards pathological states and protective factors preventing people from gravitating towards disordered states. In the case of EDs, *orthorexia* may represent such a robustly unstable state: even if it’s not part of the standard DSM taxonomy, it is plausible to think about it as a nearby state on the brink between pathological and healthy ones. See also Serpico and Petrolini (2023).

8 Bifurcation events are currently being discussed in environmental sciences: they occur when the effects of a small perturbation are amplified dramatically, destabilizing a system’s dynamics (Lenton 2020, 2025). More generally, these cascades can be seen as peculiar causal structures, often neglected in philosophy of science due to extensive focus on compositional mechanisms (Ross 2022).

9 This is also consistent with epigenetic findings (e.g., DRD2 hypermethylation in AN but not BN, see Frieling et al. 2010; Hübel et al. 2019), which suggest that restriction leaves more persistent molecular imprints on dopaminergic reward regulation. These changes may heighten vulnerability to compulsive behaviors once nutritional rehabilitation begins, but the reverse – reversing these methylation patterns through disinhibition – is less likely.

10 Consider addiction as a further example: neurobiological literature shows that changes in the brain due to substances have a particularly strong impact on developing brains and can persist long after substance use has been interrupted (U.S. Department of Health and Human Services 2016: 45, 64). Social dynamics can also influence the exacerbation or interruption of addiction. At later stages, consumption can interfere with relevant personal projects such as career, marriage, or parenthood: in these cases, some people are able to quit substance abuse when life demands them to make a choice. But in other cases, life circumstances do not make quitting a better alternative: either rehab is not a palatable option, because one’s social network incentivizes addictive behavior, or quitting drugs is not an option at all because it would not open up new significant opportunities (Castillo and Resurreccion 2019; Pickard 2021; Sheedy & Whitter 2013).

11 Interestingly, the age of onset for EDs has been steadily decreasing (for a review, see Favaro et al. 2019). However, at the same time, there is renewed interest for the study of AN in middle and older age, albeit often connected to lack of timely diagnosis or misdiagnosis in the earlier life trajectories of these individuals (Ellin 2025).

12 Not everyone agrees that biological processes involved in EDs are difficult to reverse, especially when we consider molecular levels. Literature in epigenetics often suggests that epigenetic markers, like DNA methylation, are reversible and dynamically respond to environmental stimuli. In the context of EDs, for instance, Booij and Steiger (2020) make the case that malnutrition-induced epigenetic changes in AN are reversible. Nevertheless, these molecular changes often track complex dynamics well beyond just molecular levels: epigenetic markers are thus sensitive to both ‘positive’ and ‘negative’ feedback. In our view, reversing epigenetic markers is also subject to patients’ inner feelings, self-narratives, values, and the life trajectories they contribute to shape.
